# Recurrent urinary tract infections and complications after symptomatic versus antibiotic treatment: follow-up of a randomised controlled trial

**DOI:** 10.3205/000228

**Published:** 2016-02-10

**Authors:** Jutta Bleidorn, Eva Hummers-Pradier, Guido Schmiemann, Birgitt Wiese, Ildikó Gágyor

**Affiliations:** 1 Institute for General Practice, Hannover Medical School, Hannover, Germany; 2 Department of General Practice and Family Medicine, University Medical Center, Goettingen, Germany; 3 Institute for Public Health and Nursing Research, Department for Health Services Research, University of Bremen, Germany

**Keywords:** urinary tract infections, recurrent UTI, pyelonephritis, follow-up

## Abstract

**Background:** Uncomplicated urinary tract infections (UTI) are common in general practice, and are usually treated with antibiotics. Recurrent UTI often pose a serious problem for affected women. Little is known about recurrent UTI and complications when uncomplicated UTI are treated without antibiotics. With ICUTI (Immediate vs. conditional antibiotic use in uncomplicated UTI, funded by BMBF No. 01KG1105) we assessed whether initial symptomatic treatment with ibuprofen could be a treatment alternative for uncomplicated UTI. The presented analysis aims to assess the influence of initial (non-)antibiotic treatment on recurrent UTI rates and pyelonephritis after day 28 up to 6 months after trial participation.

**Methods:** This study is a retrospective long-term follow-up analysis of ICUTI patients, surveyed telephonically six months after inclusion in the trial. Recurrent UTI, pyelonephritis or hospitalizations were documented. Statistical evaluation was performed by descriptive and multivariate analyses with SPSS 21.

**Results:** For the six months follow-up survey, 386 trial participants could be contacted (494 had been included in ICUTI initially, 446 had completed the trial). From day 28 until 6 months after inclusion in ICUTI, 84 recurrent UTI were reported by 80 patients. Univariate and multivariate analyses showed no effect of initial treatment group or antibiotic treatment on number of patients with recurrent UTI. Yet, both analyses showed that patients with a history of previous UTI had significantly more often recurrent UTI. Pyelonephritis occurred in two patients of the antibiotic group and in one patient in the non-antibiotic group.

**Conclusion:** This follow-up analysis of a trial comparing antibiotic vs. symptomatic treatment for uncomplicated UTI showed that non-antibiotic treatment has no negative impact on recurrent UTI rates or pyelonephritis after day 28 and up to six months after initial treatment. Thus, a four week follow-up in UTI trials seems adequate.

## Zusammenfassung

**Hintergrund:** Unkomplizierte Harnwegsinfekte (HWI) sind eine häufige Erkrankung in der Allgemeinmedizin und werden üblicherweise antibiotisch behandelt. Rezidive stellen dabei ein ernstzunehmendes Problem für betroffene Frauen dar. Über Rezidive und Komplikationen nach nicht-antibiotischer Behandlung unkomplizierter Harnwegsinfekte ist wenig bekannt.

Mit der Studie ICUTI (Immediate vs. conditional antibiotic use in uncomplicated UTI, gefördert vom BMBF 01KG1105) wurde untersucht, ob eine initial symptomatische Behandlung mit Ibuprofen eine alternative Behandlungsmöglichkeit beim Harnwegsinfekt darstellt. Die hier vorgestellte Analyse hatte zum Ziel, den Einfluss einer initialen (nicht)antibiotischen Therapie auf das Auftreten von Rezidiven und Pyelonephritiden zwischen Tag 28 und 6 Monaten nach Einschluss in die Studie darzustellen.

**Methode:** Retrospektive Analyse der 6-Monats-Nachbefragung der ICUTI-Teilnehmer. Durch telefonische Befragung 6 Monate nach Einschluss in die Studie wurde das Auftreten von Rezidiven, Pyelonephritiden und Krankenhausaufnahmen dokumentiert. Die statistische Auswertung erfolgte durch deskriptive und multivariate Analysen mit SPSS 21.

**Ergebnisse:** Bei der 6-Monats-Nachbefragung wurden 386 ICUTI-Teilnehmerinnen erreicht (494 waren in ICUTI eingeschlossen worden, 446 hatten die Studie beendet). Zwischen Tag 28 und 6 Monaten nach Einschluss in ICUTI berichteten 80 Patientinnen insgesamt 84 Rezidive. In der univariaten und in der multivariaten Analyse zeigte sich kein Einfluss der initialen Behandlungsgruppe oder einer antibiotischen Behandlung auf die Zahl der Patientinnen mit Rezidiven. Allerdings zeigten beide Analysen, dass Patientinnen mit vorherigen Harnwegsinfekten signifikant häufiger weitere Rezidive bekamen. Pyelonephritiden traten bei zwei Patientinnen in der antibiotisch behandelten Gruppe und bei einer Patientin in der nicht antibiotisch behandelten Gruppe auf.

**Schlussfolgerung:** Die Follow-up-Analyse der Nachbefragung nach einem Vergleich einer sofortigen antibiotischen mit einer zunächst symptomatischen Therapie des unkomplizierten Harnwegsinfektes ergab, dass eine nicht antibiotische Therapie keinen Einfluss auf das Auftreten von Rezidiven oder Pyelonephritiden zwischen Tag 28 und 6 Monaten nach der initialen Therapie hat. In weiteren Harnwegsinfektstudien scheint daher eine Nachbeobachtung von vier Wochen ausreichend.

## Background

Urinary tract infections (UTI) are a widespread disease with a lifetime prevalence of 37%, and a high risk of recurrent UTI as the majority of women (79%) report more than one infection [1]. Women affected from recurrent UTI perceive this as a burden, and many appreciate a non-antibiotic treatment approach [2]. Usually, UTI are treated with antibiotics: about 71% of women seeking a health professional receive a prescription. Prescription rates are influenced by symptom severity and patients’ age [1] and account for a significant number of antibiotic prescriptions in general practice [3], [4]. Pyelonephritis may occur as a complication of UTI, with symptoms such as fever, loin tenderness in addition to urgency, frequency and pain [5].

Little is known about recurrence rates and complications when UTI are treated without antibiotics. Up to now, some trials have assessed antibiotic treatment vs. placebo or delayed prescription [6], [7], [8], but data about recurrent UTI are hardly comparable due to different definitions. As for the risk of pyelonephritis after non-antibiotic treatment of UTI, data is sparse as well: only few trials reported data on pyelonephritis [6], [9], [10]. 

The follow-up of participants in those trials lasted from two weeks [6] up to longer than a year [8]. However, it is unclear how long patients should be followed up in UTI trials with non-antibiotic treatment approaches of UTI to follow-up recurrent UTI or complications.

With ICUTI (Immediate vs. conditional antibiotic use in uncomplicated UTI) we performed a double blind randomized controlled trial (RCT) to evaluate whether initial symptomatic treatment with ibuprofen could be a treatment alternative for uncomplicated UTI [9]. The presented analysis of long term follow-up data aims to assess recurrent UTI and complications after day 28 up to 6 months after trial participation, related to initial treatment strategy.

## Methods

### Patients

In ICUTI, 494 women aged 18–65 and presenting with typical UTI symptoms as dysuria, frequency/urgency of micturition or lower abdominal pain were enrolled in 42 general practices in Northern Germany. After informed consent, patients received a blinded trial drug which was either ibuprofen 400 mg 3x3 tbl. for 3 days, or fosfomycin 1x3 g. Urine cultures were performed from freshly voided urine to confirm infection and warrant specific antibiotic treatment in case of persisting or worsening symptoms. Key exclusion criteria were UTI within the last two weeks, antibiotic treatment within the last two weeks, current treatment with NSAIDs, fever, upper tract infection, history of gastrointestinal ulcers, severe conditions, pregnancy, renal diseases. Symptom course, antibiotic prescriptions, adverse events, complications and recurrent UTI were recorded by telephone interviews up to day 28 [9], [11].

In the additional long term follow-up study, patients were called by academic study nurses after six months and asked whether they had had recurrent UTI, pyelonephritis or hospitalizations since the last telephone call at day 28 (Table 1 (Tab. 1)). Thus, the results presented are related to the time period from day 28 until six months after inclusion in ICUTI. 

At inclusion, participants were asked to provide separate written consent for the follow-up study. 

### Statistical analysis 

Follow-up data was collected in a web-based data-entry-system (SecuTrial^®^). Recurrent UTI were defined based on patients’ report, no further testing or medical confirmation was used. Data on complicated UTI or pyelonephritis were also collected based on patients’ information.

To assess recurrence rates in relation to initial antibiotic treatment, we analyzed data first within the treatment groups patients had been assigned to (ibuprofen and fosfomycin). Since about 33% of ibuprofen patients received antibiotic treatment within 28 days after recruitment, we split the sample in two new groups: the “antibiotic treatment” group included all patients with initial antibiotic treatment (fosfomycin) and those who were initially treated with ibuprofen but had a secondary antibiotic treatment for UTI until day 28. The “non-antibiotic treatment” group included all patients who received only ibuprofen. We further assessed age, initial bacteriuria, and history of previous UTI as potential predicting factors for recurrent UTI. 

The dependent variable of the univariate analysis was patients with at least one recurrent UTI from day 28 (last phone call) up to 6 months after inclusion. To analyze the association of the predictors for this outcome, Chi-square tests were calculated. Subsequently, a multivariate logistic regression model was applied to identify independent factors on the outcome. We included the variables patients’ age, bacteriuria at inclusion, randomization to fosfomycin, antibiotic treatment and history of previous UTI in this analysis. Analyses were performed using SPSS version 21.

## Results

In ICUTI, 446 patients (222 randomized to ibuprofen, 224 to fosfomycin) completed the study until the follow-up at day 28. In 386 patients (189 randomized to ibuprofen, 197 to fosfomycin) the six months follow-up survey could be performed, 60 patients were lost to follow-up. 

Data from the ICUTI trial regarding complications and recurrent UTI from inclusion until day 28 are shown in Table 2 (Tab. 2): From day 8 to day 15 after inclusion, the ibuprofen group showed higher rates of early relapses, whereas beyond day 15 until day 28, patients in the fosfomycin group reported more recurrent UTI [9].

Considering the time period day 28 until 6 months, 84 recurrent UTI were reported by 80 patients (Table 3 (Tab. 3)). Of these, 5.6 % were feverish. In 64%, recurrent UTI were treated by the GP, and in 17.9% by patients themselves. 

Univariate analyses showed no effect of randomization group or antibiotic treatment on number of patients with recurrent UTI between day 28 and 6 months (Table 4 (Tab. 4)). Also, the initial urine culture status showed no influence. Recurrent UTI were reported less often by patients with higher age, ranging from 28% in patients aged 18–25 years down to 9.8% in the 46–55 years group, but then rose again up to 19.7% in patients aged 56–65 years, without reaching statistical significance (Table 5 (Tab. 5)).

Patients with a history of previous UTI had recurrent UTI significantly more often during the time period day 28 until 6 months (Table 4 (Tab. 4)). These results were confirmed in the multivariate regression analysis: only a history of previous UTI showed significant influence on further recurrences (OR 7, CI [3.9–12.6], Table 6 (Tab. 6)).

One patient in the non-antibiotic group reported a pyelonephritis versus two in the group with initial antibiotic treatment (not significant), resulting in a pyelonephritis rate of 3/386 (0.8%) from day 28 until six months. Two cases were treated as outpatients, one patient was admitted to hospital. 

In total, 21/386 patients reported a hospitalization. One case was related to the urinary tract (renal colic), the remaining were due to other conditions.

## Discussion

Results of this follow-up analysis after initial (non-)antibiotic treatment of UTI within the randomized controlled trial ICUTI show that recurrent UTI rates between day 28 and 6 months are not related to antibiotic or non-antibiotic treatment of the index UTI. However, patients with a history of previous UTI had a high risk for further UTIs. Comparable results were reports by a Finnish study with 44% recurrent UTI in patients with previous UTI and 11.8% in patients without previous UTI [12]. In all, the recurrent UTI rate of 20.7% until 6 months corresponds to results from other authors [13], [14], [15]. 

Trials comparing antibiotic treatment with placebo or symptomatic treatment showed different results for recurrent UTI rates but are lacking a consistent definition of recurrent UTI: Christiaens et al. reported five cases with microbiological relapse (bacteriuria after negative culture) after antibiotic treatment and three after placebo treatment [6]. A Swiss trial comparing norfloxacin and diclofenac for UTI showed more reconsultations due to UTI symptoms in the diclofenac group ([10], only congress abstract available). However, Ferry et al. reported similar rates of clinical recurrences after antibiotic treatment (pivmecillinam) and placebo treatment, with 12–13% at seven week-follow-up [7]. In our pilot study, one patient in the antibiotic group and two in the ibuprofen group reported a relapse at day 28 [16].

Apparently, the first four weeks after trial inclusion seem to be more relevant: in ICUTI early relapses (reoccurrence of symptoms day 8–14) were more frequent in the ibuprofen group, whereas recurrences day 15–28 were reported significantly more often in the fosfomycin group (Table 2 (Tab. 2)) [9]. 

Correspondingly, the number of pyelonephritis was higher in patients with non-antibiotic treatment within the first 28 days, but not later on, as ICUTI results show [9]. 

Important limitations of this study should be mentioned: First, the exact date of recurrences was not documented. Results refer to a period of 5 months, and it is not clear at which point of time recurrent UTIs occurred exactly. However, due to the retrospective design an exact date would be difficult to obtain. Second, diagnosis of recurrent UTI and pyelonephritis was not confirmed by medical records or GPs’ opinion, but was based solely on patients’ reports at the phone interview. For pyelonephritis as a more serious complication, this means a limitation – if patients’ reports are considered unreliable, the incidence pyelonephritis may be either over- or underestimated. However, pyelonephritis is a clinical diagnosis, and patients most likely saw a physician. In further studies, data on pyelonephritis should be confirmed by physicians’ records according to defined criteria. 

For recurrent UTI this seems a minor problem, since in women with recurrent UTI history taking is the cornerstone in diagnosing UTI – when typical symptoms are present further tests are not required [17]. 

Further, there might be other influencing factors for recurrent UTI which have not been considered in our data collection (i.e. sexual activity, diabetes), since the presented analysis focused on the influence of (non-)antibiotic treatment on UTI recurrence. Since women were randomly assigned to the intervention or control group, we assume that risk factors were distributed equally.

As a minor limitation, results are only transferrable to patients up to 65 years since this was the upper age limit for ICUTI participation.

## Conclusions

This follow-up analysis of a randomized controlled trial comparing antibiotic vs. symptomatic treatment for uncomplicated UTI showed that non-antibiotic treatment had no negative impact on recurrent UTI rates or pyelonephritis more than four weeks after initial treatment. Thus, a short follow-up in UTI trials seems adequate to capture recurrent UTI and complications.

## Notes

### Competing interests

The authors declare that they have no competing interests.

## Figures and Tables

**Table 1:**
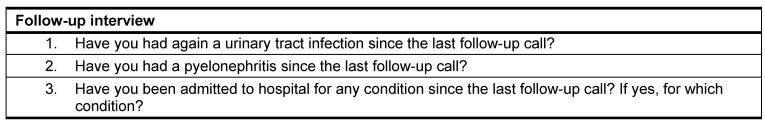
Follow-up: questions

**Table 2:**
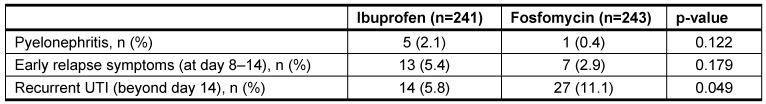
ICUTI: Recurrent UTI/complications day 0–28 (data ICUTI, intention to treat population [9])

**Table 3:**
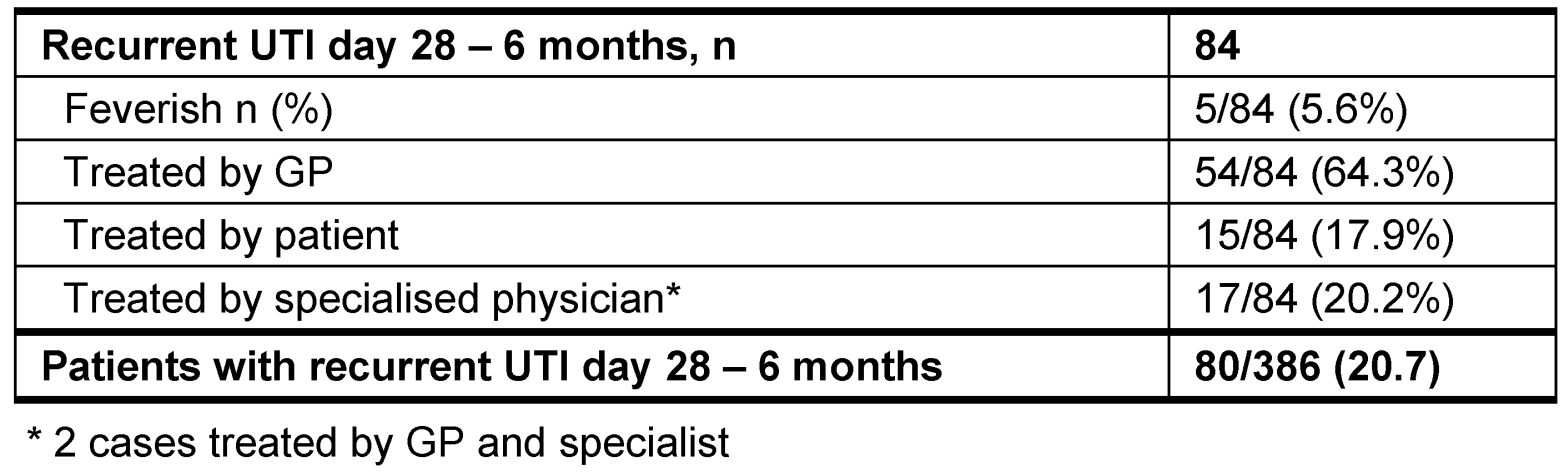
Recurrent UTI day 28 until 6 months

**Table 4:**
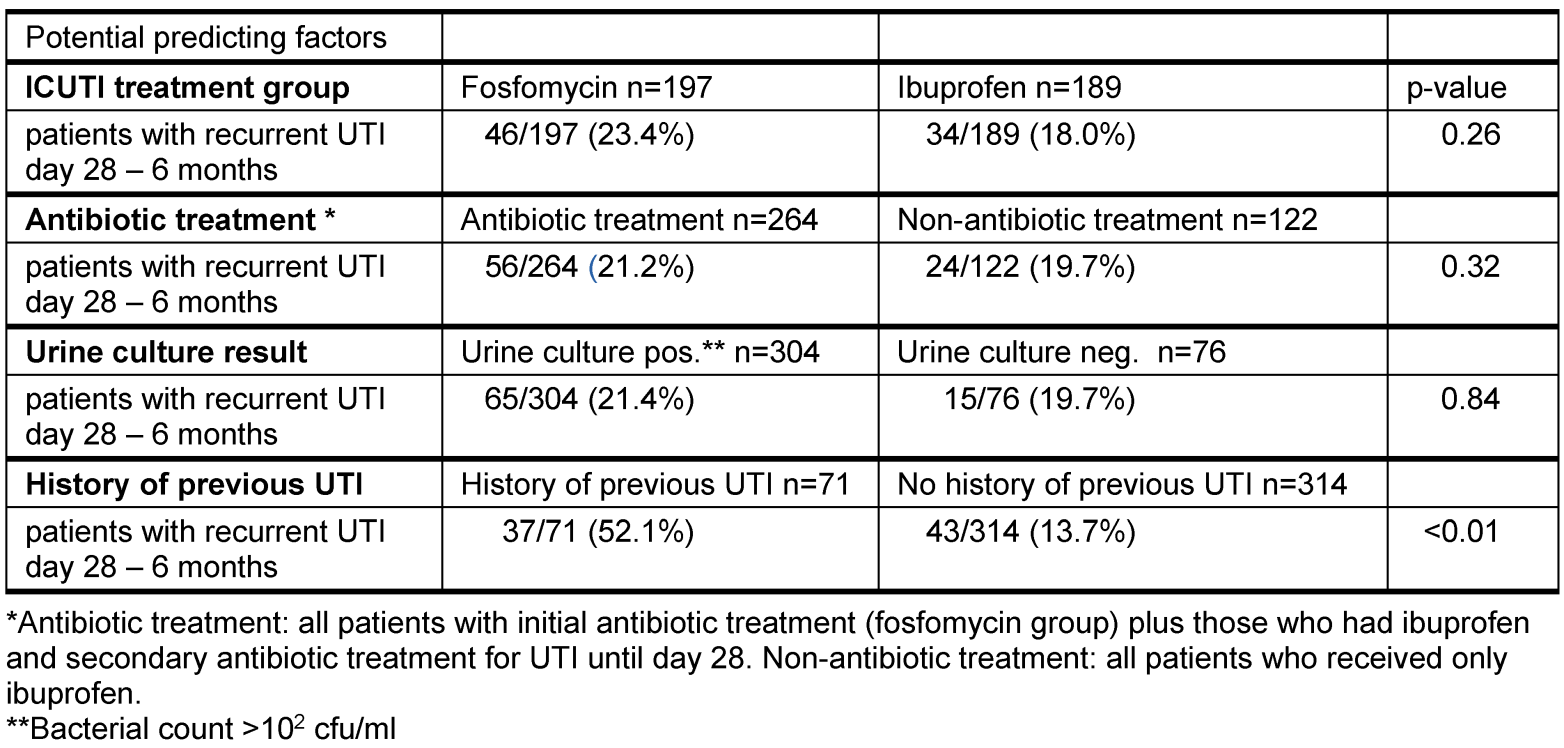
Recurrent UTI/influencing factors

**Table 5:**
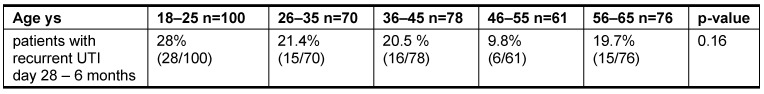
Recurrent UTI/age

**Table 6:**
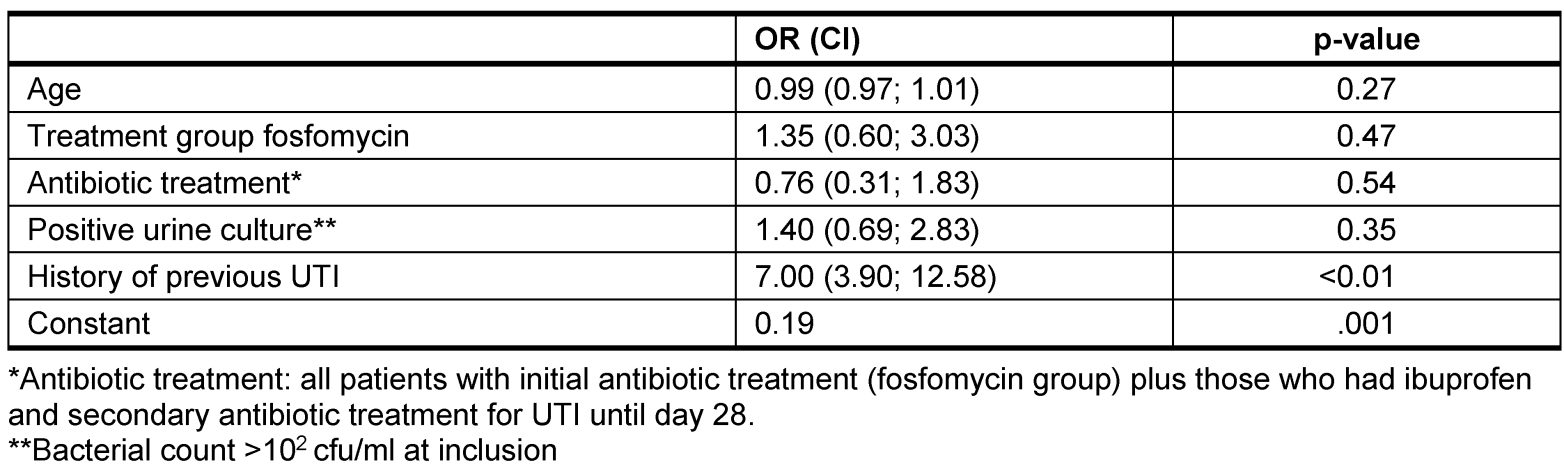
Recurrent UTI, multivariate logistic regression analysis
